# Laser spectroscopic method for remote sensing of respiratory rate

**DOI:** 10.1007/s13246-023-01292-x

**Published:** 2023-06-26

**Authors:** Wesam Bachir, Fatimah Samie Ismael, Nour Hasan Arry Alaineya

**Affiliations:** 1 Institute of Metrology and Biomedical Engineering, Faculty of Mechatronics, Warsaw University of Technology, Św. A. Boboli 8 St, 02-525 Warsaw, Poland; 2 Biomedical Photonics Laboratory, Higher Institute for Laser Research and Applications, Damascus University, Damascus, Syria

**Keywords:** Respiratory rate, Remote sensing, Laser, Spectrometer, Neonate

## Abstract

Noncontact sensing methods for measuring vital signs have recently gained interest, particularly for long-term monitoring. This study introduces a new method for measuring respiratory rate remotely. The proposed method is based on the reflection of a laser beam off a striped card attached to a moving platform simulating chest wall displacements. A wide range of frequencies (n = 35) from 0.06 to 2.2 Hz corresponding to both normal and pathological human respiratory rates were simulated using a moving mechanical platform. Reflected spectra (n = 105) were collected by a spectrometer in a dynamic mode. Fourier analysis was performed to retrieve the breathing frequency. The results show a striking agreement between measurements and reference frequencies. The results also show that low frequencies corresponding to respiratory rates can be detected with high accuracy (uncertainty is well below 5%). A validation test of the measuring method on a human subject demonstrated a great potential for remote respiration rate monitoring of adults and neonates in a clinical environment.

## Introduction

Preterm birth is a priority health issue worldwide. Breathing rate is routinely used as a vital sign for monitoring key physiological functions of the human body, particularly for the diagnosis of respiratory diseases and related abnormalities [1, 2]. Clinically, respiratory rate is generally defined as the number of inspirations observed during a minute (in breaths per minute, or bpm). Therefore, Respiratory rate can be an indicator of serious clinical events [3].

In fact, a wide range of techniques and methods can be used for recording respiratory rate, including spirometry, capnometry, and pneumographic [4–7]. Electrocardiography which records the electrical activity of the heart and photoplethysmography which reflects the volumetric changes in blood in peripheral circulation are the most used methods for estimating breathing signals in clinical settings. However, all the above-mentioned modalities are invasive techniques and sensitive to noise and artifacts. Conventional respiratory rate monitoring equipment very often involves adhesive transducers or electrodes to be directly attached to the skin. This may cause skin irritation, particularly for the preterm infant where the skin is very sensitive and fragile. There is also a risk of enabling or introducing an infection [8]. In addition to neonate discomfort, a major setback of these sensors is the likelihood of poor or loose connection of the attached sensors.

Recently, there have been other emerging methods that rely on volume changes in thoracic and abdomen areas. These changes can be detected by accelerometers, radars, Wi-Fi devices, imaging, and sensors based on electromagnetic, piezoelectric, and optical mechanisms [9]. For example, Doppler radar is a useful method because it is an accurate, non-invasive approach, and noncontact for respiratory monitoring [10]. Other researchers have investigated respiratory motion evaluation by calculating curvature variance of the chest wall using a fiber optic sensor [11] and fiber Bragg grating techniques [12]. Kim et al. [13] presented a non-contact respiration monitor that could be particularly applicable in the field of neonatal care by transmitting and receiving a band of radio waves, the IR-UWB radar system can recognize the patient’s chest movements during breathing at a distance. The developed system features many advantages offering more safety to patients who have fragile skin. In addition, radar waves do not interfere with light and have the possibility to operate in dim lighting conditions in intensive care settings. However, the radar systems can be vulnerable to noise interference from other nearby devices working in the same frequency band. Moreover, Radar-based respiratory sensing capability is limited by the presence of multiple subjects in front of the radar system and motion background [14].

Laser monitoring techniques have also been investigated featuring highly accurate measurements of chest wall displacement. However, these laser sensors [15, 16]. were bulky systems. Also, laser Doppler-based methods for estimating respiratory rate feature high accuracy, non-invasive and noncontact for respiration monitoring. Marchionni et al. [17] developed an optical measurement method based on a laser doppler vibrometer for the simultaneous assessment of respiration and heart rates. Nonetheless, the cost of such a system is remarkably high and can be unaffordable in many clinical settings. In addition, laser doppler vibrometer showed sensitivity to the surface characteristics and hence low intensity of back reflected laser [18]. Reyes et al. [19] proposed a smartphone-based respiration monitoring system for both instantaneous respiration rate estimation and tidal volume estimation via an algorithm that tracks chest movements directly from a smartphone’s camera, nonetheless, it was vulnerable to motion artifacts and required subjects to wear fitted clothes during the experiments. In another recent study, Koyama et al. [20] proposed a novel simple, and cost-effective smart textile using hetero-core optical fiber for a heartbeat and respiration monitoring, however, the developed method is inadequate for remote detection of respiration rates such as intensive care units. Thus, there is still a need for a method that can address all the challenges that face the existing methods for remote measuring and possibly long-term monitoring respiration rate in a clinical environment.

The novelty of the presented work lies in introducing a novel laser spectroscopy-based approach for directly and remotely measuring the respiratory rate that can mitigate the limitations of the existing technologies mentioned above. It is important to point out here that the principle of measurement along with the stripped card for measuring the respiratory rate has not been investigated before. Therefore, in this manuscript, we propose a simple alternative approach for measuring the respiratory rate. The noncontact spectroscopic method presented in this paper is based on the reflected laser beam from a striped card adhered to a physical model simulating the chest wall displacement of the patient. The performance of the developed system is tested and validated for a wide range of normal and abnormal respiration rates. The results demonstrate the potential capability of the presented approach in estimating respiration rate in clinical settings. A comparison with the latest technologies used in clinical or preclinical studies, including the advantages of the proposed method over the existing counterparts, is discussed.

## Materials and methods

Chest wall movement is a mechanical function of a biological system [21]. The notation b(t) can be used to represent a respiratory signal, that varies as a function of continuous time, t. Mathematically, it can be expressed as a periodic sine wave given by

1 $$b\left(t\right)={b}_{max}\text{sin}\left(2\pi ft\right)$$

where b(t) is the respiratory signal, f is the frequency measured in Hz. The frequency of interest can be determined using the Fourier transform of the measured signal denoted by B(w) expressed as

2 $$B\left(\omega \right)={\int }_{-{\infty }}^{{\infty }}b\left(t\right){e}^{-j\omega t}dt$$

**Fig. 1:**
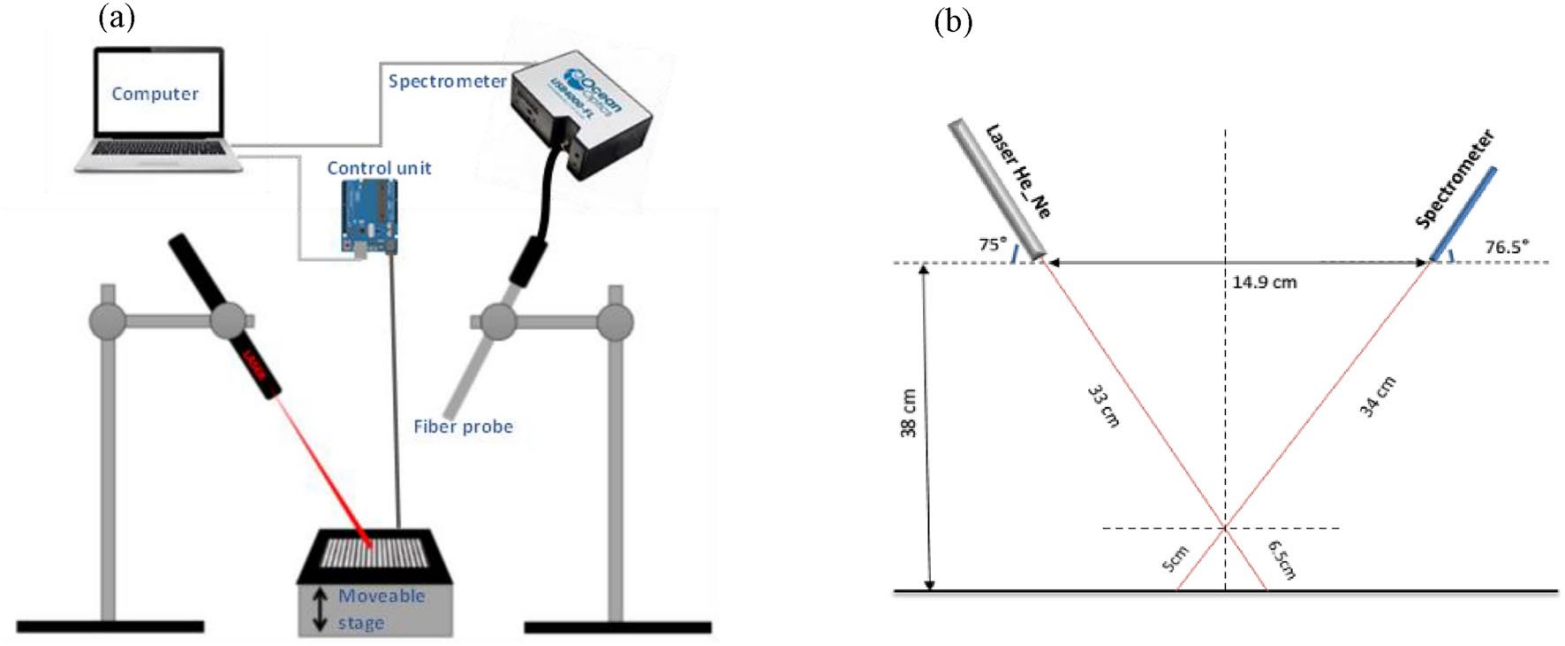
**a** Experimental setup including the movable stage on which the striped card is placed, the control unit, illumination and collection fibers, and portable spectrometer. **b** Positioning of laser and the reflection probe. The distance between the laser and spectrometer and the optical table was set to 38 cm. The laser and spectrometer were titled 15° off the vertical axis

**Fig. 2:**
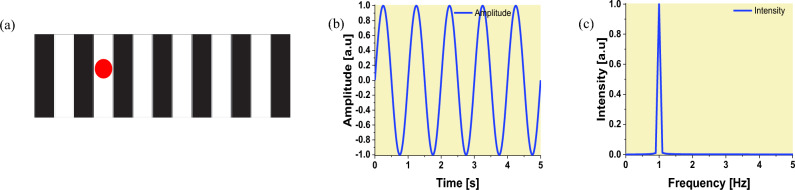
**a** The striped card with white and black bands, the laser spot is indicated by a red circle. **b** A sine wave representing the reflected laser off the vertically moving striped card. **c** The Fourier transform of the sine wave featuring the movement frequency

The experimental measuring setup used in this study is displayed in Fig. 1a. The proposed respiratory motions system consists of a vertically moving platform using a stepper motor driven by the control unit based on the Arduino UNO board. Two colour bars (black and white) are printed on a 10 cm^2^ white cartoon card (see Fig. 2). The striped card is set on the platform. While the platform is moving, the laser spot is directed to the striped card. As for the light source, a 3 mW He-Ne laser with a wavelength of 632.8 nm (25-LHP-121-230, Melles Griot, USA) was used as a light source. This laser source is considered safe if it is handled with restricted beam viewing to the eye and thus no laser protection goggles are required.

The reflected light is received by a fibre probe, (R600-7-VIS-125 F, Ocean Optics, USA) which is connected to the spectrometer (USB4000 Ocean Optics, USA). The Spectra Suit software (Ocean Optics, USA) was used for recording spectra. Dimensions and angles used for the measurements are shown in Fig. 1b.

According to Wijenayake et al. [22] and Singh et al. [19], normal adults’ chest displacement is about 1.2 cm and it can be amounted to up to 4 cm in irregular respiratory cases. Consequently, through this study, the regular chest wall displacement (step) varied between 1 and 5 cm. Fleming et al. [23] presented that the normal range respiratory rate for infants at the age (of 0–3 months) is in the range 34–57 bpm so the normal frequency range is 0.4 Hz–1 Hz.

Therefore, the height of the movable stage (see Fig. 1) can be adjusted to provide five displacements (1, 2, 3, 4, and 5 cm) that correspond to the regular and irregular chest wall displacements. These displacements are convenient to represent the chest wall motions for infants and adults. Also, the controlled movable stage allows for moving the striped card at predefined frequencies that are compatible with the range of respiratory rate (breath/minute) for infants and adults as well. In other words, for each platform displacement, the motor speed was varied and set to 7 speeds (10, 20, 30, 40, 50, 60,70 RPM (rotation per minute)). Table 1 lists the corresponding frequencies used in the experimental work.

**Table 1:** The calculated frequencies of the mechanical platform movements. These frequencies were calculated for a range of vertical displacements from 1 to 5 cm with a step of 1 cm and a range of RPM values from 10 to 70 rpm with a step of 10 rpm.

Rpm [rpm]	Step [cm]				
	1	2	3	4	5
	Frequency [Hz]				
10	0.333	0.1655	0.1101	*0.07683*	*0.06993*
20	0.6666	0.3291	0.2196	0.1703	0.1396
30	0.9876	0.4828	0.3381	0.2491	0.2115
40	*1.31*	0.6474	0.4348	0.3318	0.279
50	*1.6*	0.8046	0.5673	0.4094	0.3322
60	*1.861*	0.9584	0.6931	0.4971	0.4094
70	*2.286*	*1.111*	0.793	0.5785	0.4633

Italics values represent abnormal frequencies used to simulate the pathological respiration rates

As mentioned earlier, black and white bars were printed on the striped card. The laser spot was 2 mm in diameter on the surface of the striped card. The laser source was tilted off the vertical at an angle of 15°, to make sure that the reflected light from the striped card is within the detection cone of the collection fibre (the numerical aperture of the collection fibre is 0.22). The laser spot covered one bar on the striped card (2 mm). The initial trials regarding the bar width showed that the 2 mm bar width which equals the laser beam width was the best one.

**Fig. 3:**
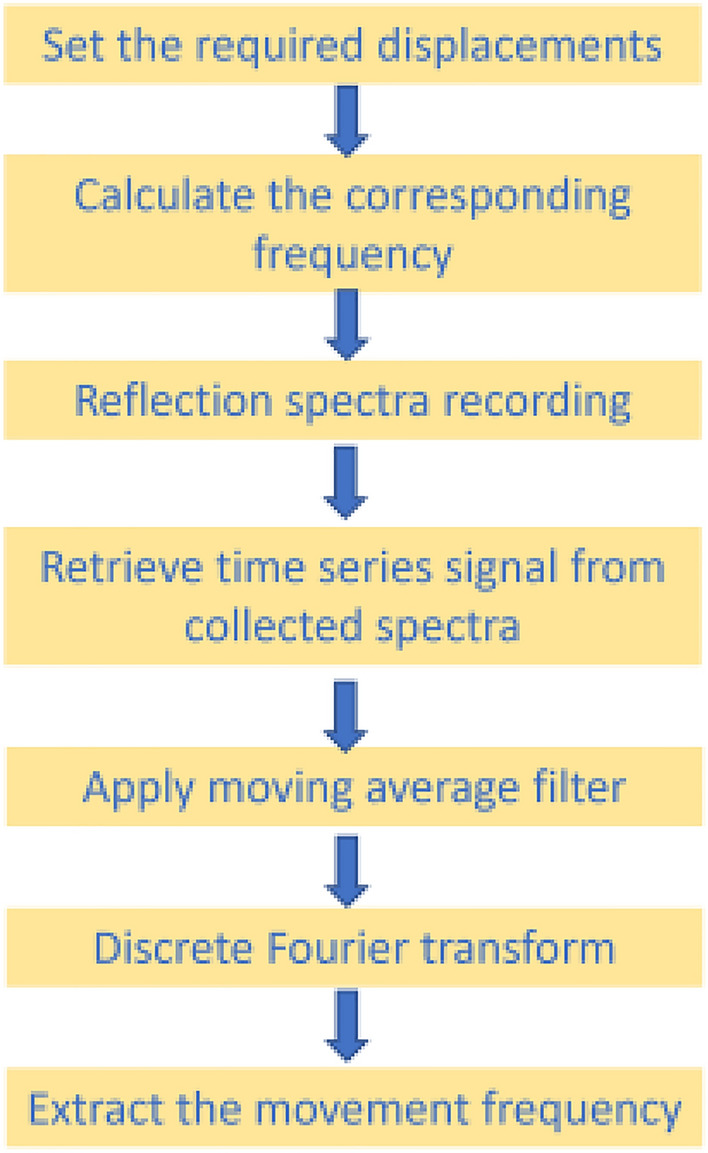
Flow chart of the experimental steps for recovery of the movement frequency of the wall chest mimicking model

Figure 3 represents the flow chart of the experimental procedure used in this work to extract the frequency of interest that resulted from the striped card displacement. The reflected spectra from the striped card were recorded with Spectrasuit software. The collected spectra were then processed in MATLAB to recover the time series signal that corresponds to the vertical displacement. To that end, another in-house MATLAB code was developed for spectral data processing particularly for saving the time-resolved measurement data in the form of a 3D data matrix (intensity-wavelength time), with subsequent intensity-time sections at the laser wavelength of 632 nm. After further processing, these sections were converted into respiration sequences. This was followed by applying a moving average filter to the time series signal for smoothing and removing high-frequency distortions in the signal. Then, the Discrete Fourier Transform (DFT) of the signal was generated and the movement frequency of interest was then extracted from the DFT signal. Statistical analyses and visualizations were performed using Origin software (Origin Lab Corporation, USA).

## Results

A total of 35 mechanical movements simulating the human chest wall displacement were created using a moving striped card. These movements were based on the proposed RPM (seven steps) and the vertical displacement (five steps) given in Table 1.

**Fig. 4:**
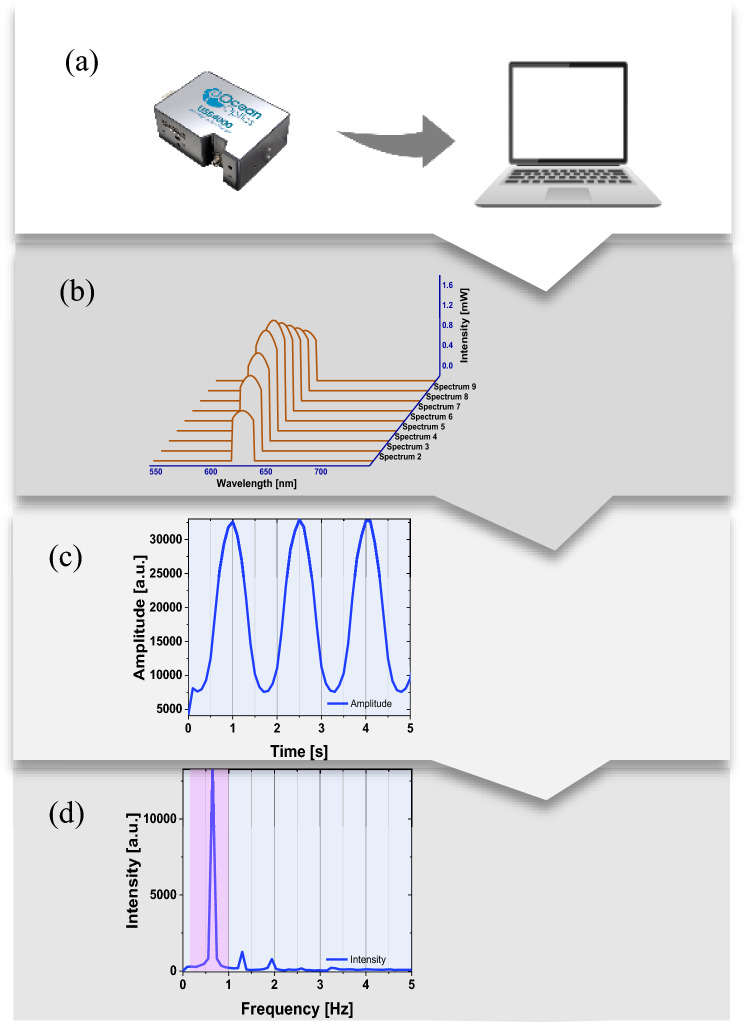
Overview of the procedure used for determining the respiratory rate **a** time-resolved spectra recorded with spectrasuit software, **b** spectral analysis to extract the time series signal corresponding to the movement under investigation, **c** the reconstructed respiratory signal, and **d** retrieval of the respiratory rate (movement frequency) using Fourier transform analysis. The shaded area on the graph indicates the normal respiration rate range i.e. 0.1 to 1 Hz

Spectra were recorded during the movement and continuously over time with a sampling frequency of 10 Hz. Given that the highest frequency in the list of predefined frequencies is 2.286 Hz, the sampling frequency of the developed system used in this work was well above the highest frequency of interest. This is in line with the Nyquist theorem. The measurements were repeated three times for every frequency measured.

**Fig. 5:**
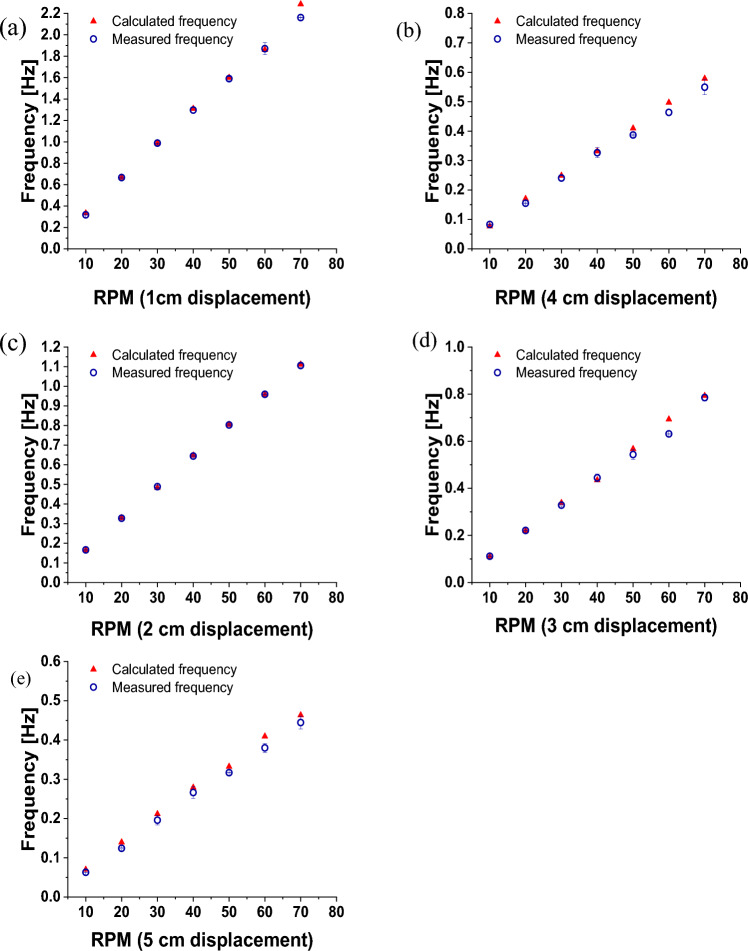
Estimated frequencies from the recorded spectra for different displacements **a**–**e** and a range of RPM values. Present frequencies (calculated) are shown for comparison

Spectral measurements were made with a portable spectrometer over nearly 1 h. Figure 4 illustrates how the respiration waveform was constructed from time-resolved spectral measurements of the reflected laser beam off the striped card. Then, the respiration rate can be estimated from the Fourier transform of the respiration waveform. Figure 4c shows an example of the extracted respiration rate from phantom measurements (displacement 2 cm and rpm 20). The measured frequencies were extracted using Fourier transform as shown in Fig. 4d. All measured frequencies were easily identified and determined. Figure 5a–e shows plots for the measured and predefined frequencies for five displacements. It should be noted here that the standard deviation is not visible due to its small value. The results show a good agreement between the measured and true frequencies. The accuracy of the measuring method introduced in this study can be evaluated by the average absolute uncertainty ± standard deviation. The absolute uncertainty was calculated using the following expression.

3 $$Absoliute\,uncertainty\left( \% \right) = \frac{{\left| {Estimated\,frequency - true\,frequency} \right|}}{{true\,frequency}}\,\left( {100} \right)$$

**Fig. 6:**
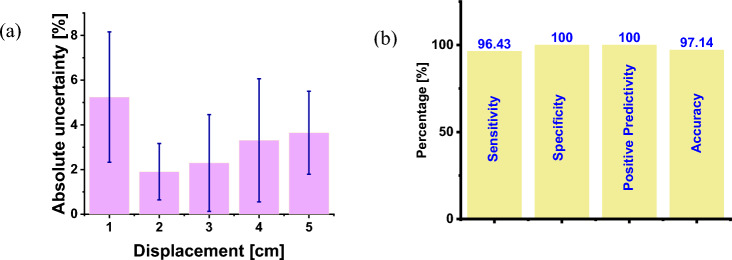
**a** Absolute uncertainty in estimating the reparation rate measurement for each of five displacements from 1 to 5 cm. **b** Sensitivity, specificity, positive predictivity, and accuracy of the respiratory rate measurements

The striking proximity between the extracted and expected frequencies indicates the feasibility of the suggested method to detect low frequencies with relatively high accuracy and an overall absolute uncertainty (considering all displacements) not exceeding 5% as shown in Fig. 6a. It can also be seen that the individual uncertainties for all displacements varied from 2 to nearly 5%.

**Fig. 7:**
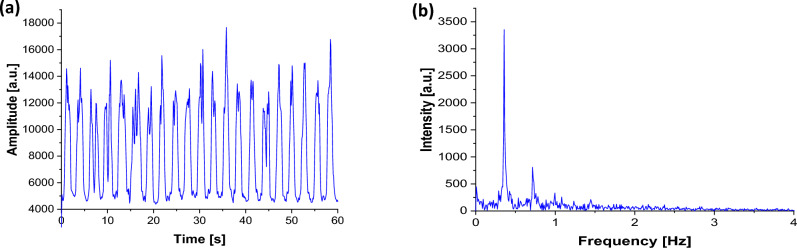
**a** The reconstructed respiratory signal of a human subject, and **b** retrieval of the respiratory rate (movement frequency) using Fourier transform analysis

The proposed method was validated with human subject measurements. The subject was a healthy non-smoking female aged 40 and in a sitting position. We used the same striped card that was used with the simulating phantom mentioned earlier in this study. The card was placed on the chest 0.5 m away from the laser source and collection fibre. Figure 7a shows a 1 min reconstructed respiration signal measured from the subject and the Fourier transform spectrum of the signal. Apparently, the detected frequency can be easily extracted from the spectrum as shown in Fig. 7b. The experiment was repeated three times and the mean value of the respiration rate frequency was 24.08 ± 2.44 bpm. The measured frequency was retrieved without any pre-processing of the time-domain signal. The overall uncertainty level of the measured respiration rate was 1.93%. The true respiration rate of the subject was calculated manually by counting the number of inspirations in 1 min and its mean value was 24 ± 2 bpm. The validation test demonstrates the potential capability of the proposed method in measuring the respiration rate.

## Discussion

This study demonstrated the capability of using a reflected laser beam together with a striped card for detecting the frequency of a moving object. More importantly, the experimental measurements of the frequencies with the aid of a portable spectrometer were reliable. Furthermore, the average overall uncertainty for all displacements considered in this work was found to be well below 5%. This uncertainty level is comparable to existing respiratory monitors [24]. As a result, precise measurement of the frequency can be attained.

The presented results are significant in several respects. First, the proposed approach is very simple as it relies only on the reflected laser beam. The finding of this work has also revealed the possibility of constructing a more compact device for detecting the respiration signal from the human subject. Applying the same technique can be carried out using a compact laser diode with a proper detector operating at a specific wavelength possibly in the near-infrared region so that the process of recording time-resolved spectra can be avoided provided that the detector has good sensitivity and fast response that meet the requirements of biomechanical displacement of the chest wall. The illuminating and collection components can be packaged in one single unit for easy positioning above the human subject of interest for both adults and neonates. The possibility of having a compact size of the proposed system can be seen as another strength of the developed approach in comparison with bulky existing devices such as laser vibrometers. In addition, the compact packaging allows for the proposed approach to be realized inside the incubator for infant monitoring.

Second, the remote and noncontact suggested technique is an added value to adult and infant patients since it offers an enhanced modality over the existing contact methods. Third, the results presented earlier demonstrate the remarkable accuracy of this method in detecting the reparation rate. More specifically, with spectral measurements carried out in this work, a submillimetre accuracy can be anticipated which is more than sufficient for remote real-life reparation rate measurements. Accurate measurement down to ~ 1 mm corresponding to human chest wall movement is still a big challenge for the state-of-the-art imaging sensor used for detecting respiratory rates in adults and neonates [25, 26]. In other words, our proposed laser spectroscopic technique outperforms the exiting high-resolution time of flight camera-based system with which the depth resolution is around several millimetres for short range measurements.

As for the pre-set frequencies used for evaluating the proposed method, seven frequencies out of the 35 pre-set frequencies listed above can be considered abnormal frequencies representing different medical conditions or disorders. Figure 6b displays the diagnostic evaluation parameters of the developed system. The result’s sensitivity, specificity, positive predictivity, and accuracy demonstrate the remarkable performance of the devised laser respiratory detector, particularly for the discrimination between normal and pathological respiratory rates.

Moreover, the low and safe laser power used in this work allows for continuous long-term monitoring of the breathing movement of adults and children in intensive care units and contributes to the detection of the causes of breathing-related disorders.

The present work also demonstrates the applicability of using the mechanical platform for simulating chest movement. In Previous studies, the simulated respiration signal was frequently generated by a speaker membrane [27]. In contrast, in this research, the movement of the chest is simulated by a mechanical stage which resembles to a large extent the real chest wall movement making it a more realistic simulator, particularly for large displacements.

In comparison with similar works in the literature, Joshi et al. [28] study, for instance, aimed to extract respiratory waves from the BSG and CI signals and not to detect the exact frequency of neonates. Their method asserts that the respiratory rate per minute can be detected from either the BSG or CI signals where the correlation is 0.74. The resulting RMSE value was more than 5 and the study included 10 preterm infants only. Therefore, Joshi study may need a larger number of samples to obtain more accurate values. The importance of Joshi et al. method is that it included children with lightweight where the chest movement is of low amplitude. This is in agreement with our study as the displacement of the vertical card can be smaller and up to 1 cm. The importance of the Kim et al. study [29] is that it is non-contact and depends on the IR-UWB radar system which has high accuracy, but it required more complex equipment compared to the equipment we used in this work.

Unlike other methods reported previously, a remarkable advantage of our developed detector lies in its simplicity both in hardware and software in comparison with the remote respiratory estimator introduced recently. Most of them require computationally demanding RGB image processing [30] or depth imaging analysis to estimate the respiration rate [31]. In addition, a number of recent studies have reported the dependency of the remote respiration rate measurements on the interaction between light and the skin such as age, gender, skin tone, and obesity to name a few [32]. In contrast, our system has the advantage of not being affected by individual patient variation. This would allow for skin-independent and higher signal-to-noise ratio measurements for all subjects as the laser light is reflected off the striped card and not from the skin surface.

Moreover, it is noteworthy to say that the spectrometer used in this work is very sensitive to the movement of interest and the measured reflected beam is dependent on the lateral position of the detector relative to the illuminating laser source position. In other words, the larger lateral distance between the laser source and the collection fiber should be kept to a minimum and that would require a smaller tilting angle of the collection fiber relative to the horizontal distance between the light source and the collection fiber. Therefore, our finding suggests a smaller tilting angle to avoid any possible distorted projection of the laser beam on the striped card. Whilst other collection devices other than optical fiber can be used in practice, the numerical aperture of the collection sensor must be taken into consideration for effective detection of the desired signal.

Despite the accurate measurement of movement frequency, we have obtained in this study, there were a few limitations that may have affected the experimental work. Firstly, the laser source used in this work was a relatively old light source which resulted in a possible lack of power stability throughout experiments. Secondly, the mechanical platform for simulating the respiration signal was constructed with in-house resources, and hence not highly accurate. This may also have an impact on the accuracy of the results. Finally, the striped card used in this work was also in-house and made from some basic readily available materials. As a result, the white and black bars on the striped card lacked high homogeneity. However, the practical limitations described above did not undermine the potential applicability of the devised method for detecting respiratory signals.

Needless to say, changing the position of the striped card due to the patient’s movement can certainly affect the measurements. This could be a limitation if the card size is small thus, one way to overcome this drawback is to make the card relatively big in size. However, this should not be seen as a negative point since the body movement of the patient can be detected by the proposed device, particularly in the case of neonates. More importantly, the proposed method can also be used for instance for detecting neonatal apnea. Moreover, the proposed device could be utilized for respiration rate monitoring in intensive care units where patients are usually in sitting or lying positions with virtually no movement at all.

The results presented in this work demonstrate the feasibility of using the proposed method for low-frequency measurements corresponding to human breathing. Although the main purpose of the study was to evaluate the performance of the proposed method as the measurements were made using breathing mimicking object based on a striped card, A validation test of the proposed method on human subjects, the preliminary results presented above are promising, however, further measures need to be taken for optimal measurements in clinical settings. Long-term monitoring of respiration rate can be investigated as well. Therefore, it can be highly anticipated that the promising findings can pave the way for this method to be endeavoured for real-life clinical measurements on humans particularly neonates to explore its potential applicability for measuring the breathing rate in a clinical environment.

Overall, the proposed method possessed several other advantages that may be useful for clinical implementation. The high accuracy, sensitivity, and specificity of the suggested method indicate its outperformance over the most common methods in clinical use particularly thoracic impedance, and electrocardiographic methods [33]. Furthermore, recent wearable technology has been extensively used for measuring the respiratory rate suffer from being not accurate due to the indirect approach to estimating the respiratory rate. In addition, the proposed technique exhibits higher accuracy compared to wearable gadgets such as smart watches as their accuracy are highly dependent on the inherent signal processing techniques. Wearable technology can also be sensitive to body movements and need to be worn or placed on the body surface making them invasive and inappropriate for hospital settings or neonatal wellbeing monitoring.

Moreover, the proposed method uses a laser beam that is potentially less susceptible to environmental noise, compared to, for example, acoustic or electrical methods. Safety of the proposed optical method can be of paramount importance since the use of other existing techniques that require the radiation of microwaves such as radar-based systems for long term infant monitoring can be a matter of concern. Therefore, the suggested method provides a safe long-term monitoring of infants in comparison with the latest radar-based systems found in the literature. It must be also noted here that unlike other research instrumentation or developed devices which are either bulky or require sophisticated signal processing and analysis to obtain reasonably accurate respiratory rate, our proposed system using laser spectroscopy is very simple regarding the hardware and software to extract the respiratory rate with high accuracy. This indicated that our method is cost effective in comparison with other technologies under investigation. Consequently, our proposed method tackled all the technological challenges regarding the invasiveness, accuracy, safety, suitability for adults and neonates, susceptibility to motion artifacts and ambient noise, by offering a more convenient approach for remote, non-invasive, highly accurate, and contactless respiratory rate measurements.

## Conclusion

This research attempted to characterize a novel, low-cost, and accurate system for measuring the respiration rate resulting from chest wall displacement in adults and children. The developed breathing rate measuring system offers a simple, noninvasive, noise-free, and accurate solution. Therefore, the proposed system represents an important step toward the development of a compact system for remote long-term respiration monitoring in intensive care units. Further clinical studies can be conducted to evaluate the diagnostic protentional of the developed technique in a clinical environment.

## Data Availability

Data are available upon reasonable request.
